# Establishment and characterization of a novel multidrug-resistant pancreatic ductal adenocarcinoma cell line, PDAC-X1

**DOI:** 10.1186/s12885-024-12588-w

**Published:** 2024-07-04

**Authors:** Cheng Yu, Yuanhui Su, Xin Miao, Changpeng Chai, Huan Tang, Lu Li, Jianfeng Yi, Zhenzhen Ye, Hui Zhang, Zhao Hu, Luyang Chen, Ning Li, Hao Xu, Wence Zhou

**Affiliations:** 1 The Second Clinical Medical School, Lanzhou University, Lanzhou, 730000 China; 2 Department of Anesthesiology, Lanzhou University Second Hospital, Lanzhou, 730000 China; 3 The First Affiliated Hospital of Zhejiang Chinese Medical University, Zhejiang Provincial Hospital of Chinese Medicine, Zhejiang Chinese Medical University, Hangzhou, 310006 China; 4 The First School of Clinical Medicine, Zhejiang Chinese Medical University, Hangzhou, 310006 China; 5 The Fourth Department of General Surgery, The First Hospital of Lanzhou University, Lanzhou, 730000 China; 6 The First Clinical Medical School, Lanzhou University, Lanzhou, 730000 China; 7 The First School of Clinical Medicine, Gansu University of Chinese Medicine, Lanzhou, 730000 China; 8 Department of General Surgery, Lanzhou University Second Hospital, No. 82 Cuiyingmen, Chengguan District, Lanzhou, 730000 China; 9 Department of Hepatobiliary Surgery, Zhejiang Provincial Hospital of Chinese Medicine, The First Affiliated Hospital of Zhejiang Chinese Medical University, Hangzhou, 310006 China

**Keywords:** Pancreatic cancer, Tumor cell line, Chemotherapy, Intrinsic multidrug resistance, Transplanted tumor

## Abstract

Drug resistance remains a significant challenge in the treatment of pancreatic cancer. The development of drug-resistant cell lines is crucial to understanding the underlying mechanisms of resistance and developing novel drugs to improve clinical outcomes. Here, a novel pancreatic cancer cell line, PDAC-X1, derived from Chinese patients has been established. PDAC-X1 was characterized by the immune phenotype, biology, genetics, molecular characteristics, and tumorigenicity. In vitro analysis revealed that PDAC-X1 cells exhibited epithelial morphology and cell markers (CK7 and CK19), expressed cancer-associated markers (E-cadherin, Vimentin, Ki-67, CEA, CA19-9), and produced pancreatic cancer-like organs in suspension culture. In vivo analysis showed that PDAC-X1 cells maintained tumorigenicity with a 100% tumor formation rate. This cell line exhibited a complex karyotype, dominated by subtriploid karyotypes. In addition, PDAC-X1 cells exhibited intrinsic multidrug resistance to multiple drugs, including gemcitabine, paclitaxel, 5-fluorouracil, and oxaliplatin. In conclusion, the PDAC-X1 cell line has been established and characterized, representing a useful and valuable preclinical model to study the underlying mechanisms of drug resistance and develop novel drug therapeutics to improve patient outcomes.

## Introduction

Pancreatic cancer (PDAC) is an aggressive and deadly cancer with a 5-year survival rate of 5–10%. It ranks as the 7th most common cancer in the world, with a gradually increasing incidence rate. Surgical resection followed by adjuvant chemotherapy remains the only potentially curative treatment option. However, due to challenges in detection, the majority of patients are diagnosed with advanced disease, resulting in a low surgical resection rate [1]. Standard adjuvant chemotherapy faces challenges due to the emergence of intrinsic and acquired resistance, leading to failure of therapy and cancer relapse [2–4].

Multiple mechanisms are involved in the development of tumor resistance, including alterations in anticancer drug targets, activation of signaling pathways, changes in the tumor microenvironment (TME), DNA damage repair, epithelial-mesenchymal transition (EMT), and changes in cellular pharmacology. By understanding these mechanisms, clinicians can optimize treatment approaches to overcome or prevent drug resistance [5–7].

Drug-resistant cell lines are the most commonly used model to investigate mechanisms of drug resistance. However, given the diverse etiological causes of PDAC, including ethnic-related genetic aberrations and other variables [8–12], it is crucial to employ appropriate preclinical models that reflect these characteristics.

Here, a new Chinese pancreatic cancer cell line, PDAC-X1, has been established that exhibits intrinsic multidrug resistance. This cell line can be utilized to study the underlying mechanisms of drug resistance of PDAC, identify novel drug targets, and improve outcomes for PDAC patients.

## Materials and methods

### Tissue source

**Fig. 1:**
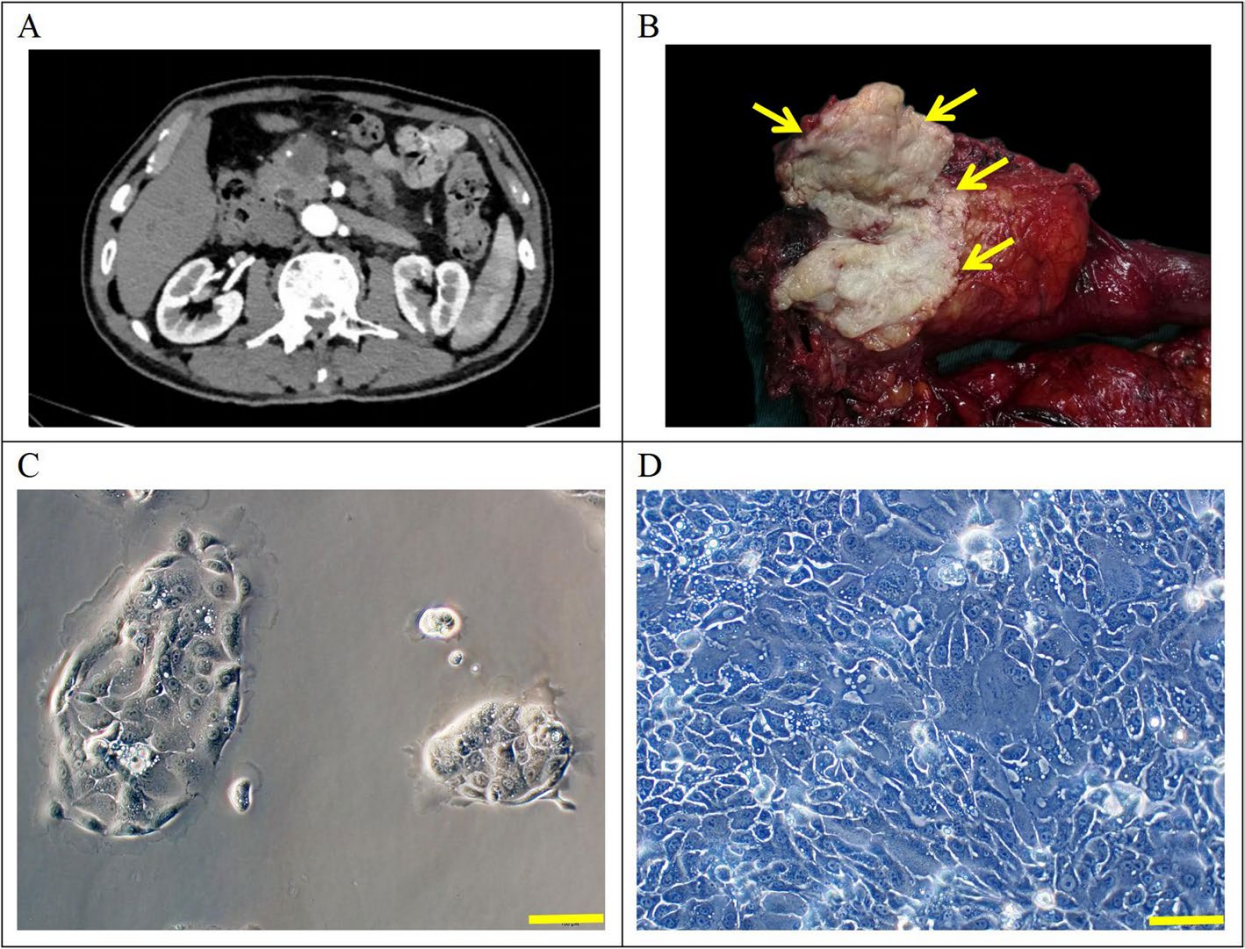
**A** Preoperative CT showing a 3 × 2 cm mass in the head of the pancreas, with weaker enhancement intensity in the arterial phase compared to surrounding normal pancreatic tissue; **B** Gross view of the specimen with a 3 × 2 cm grayish-white mass in the head of the pancreas(yellow arrow); **C**-**D** Morphology of PDAC-X1 cells. C: Morphology at low density. D: Morphology at high density. Scale bar = 100 μM

The patient was a 70-year-old male who presented with upper abdominal pain and bloating, with no history of smoking or alcohol consumption. Initial admission laboratory tests showed levels of CEA 4.3 ng/ml (reference range: 0–5.2 ng/ml), AFP 2.7 U/ml (reference range: 0–5.8 U/ml), CA19-9 82.9 U/ml (reference range: 0–35 U/ml) (Table 1). A preoperative CT revealed malignant tumors in the head of the pancreas (Fig. 1A).

**Table 1:** Clinical data of the included patient

Cell line	Patient age/ethicity	Gender	Current Status (days)	Histopathology/ differentiation	Tumor size(cm)	Prior therapy	Culture date	Serum		
								AFP IU/ml (0–5.8)	CEA ng/ml (0–5.2)	CA19-9 U/ml (0–35)
PDAC-X1	70/Asian	Male	Alive	Poorly	3 × 2	None	2022–5-20	2.7	4.3	82.9

On May 20th 2022, the patient underwent pancreaticoduodenectomy at Lanzhou University Second Hospital. The surgical specimen showed a 3 × 2 cm gray-white, hard tumor in the head of the pancreas (Fig. 1B). Tissue samples taken from the primary lesion were isolated to establish a cell line.

This study was approved by the Medical Ethics Committee of Lanzhou University Second Hospital (2023A-381), and the patient signed an informed consent form.

### NXG mice

Three female NXG mice, aged 5–6 weeks and weighing 11–15 g, were used as experimental animals. The mice were purchased from Changzhou Cavens Experimental Animal Co., Ltd. and housed in the SPF-level laboratory of the Animal Experimental Center at Lanzhou University. The animal protocol was designed to minimize pain and discomfort to the animals. For 2 weeks before experimentation, animals were acclimatized to laboratory conditions (23℃, 12 h/12 h light/dark, 50% humidity) with ad libitum access to autoclaved rodent food and water. The bedding, food, and water were replaced every 2 days. All procedures followed the institutional and national guidelines for the care and use of laboratory animals. Animal health and behavior were monitored daily for 4 weeks. When the maximum diameter of the xenograft tumor was approximately 1.5 cm or after 1 month of xenograft growth, the mice were euthanized by barbiturate overdose (intravenous injection, 150 mg/kg pentobarbital sodium), confirmed by loss of heartbeat, breathing, and pupil response.

Animal experiments were reviewed and approved by the Medical Animal Experiment Ethics Committee of Lanzhou University Second Hospital (D2023-318). The animal experiment was conducted in accordance with the ARRIVE guidelines and the Guidelines for the Care and Use of Laboratory Animals of China.

The methods in this study were similar or identical to those employed in previous studies [13, 14].

### Primary culture, cell purification, and cell line establishment

The tumor tissue was washed in sterile phosphate buffered saline (PBS) (Gibco) 3–5 times. The washed tissue was cut into small pieces using a sharpened blade and transferred into a type II Collagenase (Gibco) and Dispase (Gibco) mixture and digested enzymatically in a shaking table at 37 ℃. The supernatant was extracted when the tissue block was halfway digested, filtered with a 100 mesh filter, centrifuged at 300* g* for 3 min, and discarded. The precipitate was resuspended in PBS, centrifuged at 300* g* for 3 min, and mixed with complete culture medium (RPMI-1640 + 10% FBS + 1% penicillin–streptomycin, BI). The suspension was inoculated into a six-well plate (NEST). Fibroblasts were removed using a mechanical scraping method. Cell growth status was regularly monitored under an optical microscope. Starting from the third generation, the cells were passaged in a 1:2 ratio and frozen in serum-free rapid cell cryopreservation solution (Mei5 Biotechnology).

### Analysis of DNA short tandem repeat sequences

PDAC-X1 cells in the logarithmic growth phase were digested with trypsin, centrifuged, and analyzed with STR along with the primary tumor tissue by Suzhou Genetic Testing Biotechnology Company to understand the correlation between the cells and the primary tumor tissue.

### Cell growth curve

PDAC-X1 cells in the logarithmic growth phase were prepared as a single-cell suspension by enzymatic dissociation with 0.25% trypsin (VivaCell). After cell counting, 8 × 10^3^, 1 × 10^4^, 1.2 × 10^4^, 1.5 × 10^4^, 1.8 × 10^4^, 2.1 × 10^4^, 2.4 × 10^4^, and 2.8 × 10^4^ cells were inoculated into 9 wells of a 96-well plate (NEST). The liquid volume in each well was supplemented to 100 μl using complete culture medium. After 20 h, 100 μl diluted CCK8 solution [10% CCK8 (APE x BIO) + 90% RPMI-1640 (Gibco)] was added. After 3.5 h, the absorbance was measured at 450 nm using an enzyme-linked immunosorbent assay (BioTek, synergy H1), and a standard curve was plotted. Population doubling time were computed with several time points, using the “cell calculator +  + ” tool [V. Roth MD, Doubling Time Calculator (2006), https://doubling-time.com/compute_more.php].

### Chromosome analysis

PDAC-X1 cells in the logarithmic growth stage were treated with 0.2 ug/ml colchicine (Spectrum) and incubated for 90 min. The cells were digested using 0.25% trypsin (Vivacell), centrifuged, and the supernatant was discarded. The precipitate was resuspended in a 0.56% KCl solution and incubated at 37 ℃ for 30 min. To obtain the chromosome suspension, the cells were fixed in formic acid with glacial acetic acid (3:1), mixed well, centrifuged, and the supernatant was discarded. One drop of the suspension was placed onto a glass slide and dried in an 80 ℃ oven for 3 h. Trypsin was added to the dried glass slide for 1 min, then stained in BIOSIC solution for 8 min. The solution was removed and the glass slide was rinsed with running water. The chromosomes were observed under a 100 × oil immersion microscope. Karyotype analysis was performed using Image J imaging software and Chromosome J plugin.

### Organoid culture from PDAC-X1 cells

PDAC-X1 cells in a logarithmic growth phase were subjected to digestion followed by centrifugation. The cells were washed twice with PBS and resuspended in complete culture medium (RPMI-1640 with 10% FBS and 1% penicillin–streptomycin, BI). The resuspended cells were seeded onto an ultra-low attachment six-well plate (Corning) at a density of 1,000 cells per well, and each well was supplemented with 2 ml of culture medium for propagation. The growth state and the number of organoids were regularly inspected under a phase-contrast microscope.

### Scanning electron microscopy

PDAC-X1 cells in the logarithmic growth phase were enzymatically dissociated, centrifuged, and then inoculated onto sterile slides. When the cells were confluent over 2/3rd of the slides, the culture medium was discarded and the slides were washed with PBS (Vivacell). A 2.5% glutaraldehyde fixing solution (Servicebio) was added and fixed at room temperature in the dark for 30 min. Cells were stored and transported at 4 ℃, then sent to Wuhan Saiweier Biotechnology Co., Ltd. for embedding, staining, and subsequent processing. Finally, the prepared samples were observed and images were captured using a scanning electron microscope (HITACHI, Regulus 8100).

### Transmission electron microscopy

The culture medium was discarded from PDAC-X1 cells in the logarithmic growth phase, then 2.5% glutaraldehyde fixative (Servicebio) was added for 5 min. Fixed cells were scraped from the culture dish with a cell scraper and centrifuged. The glutaraldehyde fixative was discarded, then the new glutaraldehyde fixative was added and fixed at room temperature in the dark for 30 min. Cells were stored and transported at 4 ℃, then sent to Wuhan Saiweier Biotechnology Co., Ltd. for embedding, ultra-thin sectioning, and staining steps. Finally, the prepared samples were observed and images were captured using a transmission electron microscope (HITACHI, HT7800).

#### Drug sensitivity experiment

PDAC-X1 cells in the logarithmic growth phase were taken and a single-cell suspension was prepared after enzymatic dissociation. The cell density was adjusted to 1.2 × 10^5^/ml, and 100 μl/well was added to the 96-well plate (NEST). After 24 h, anti-tumor drugs (oxaliplatin, fluorouracil, gemcitabine, and paclitaxel) were diluted into solutions of different concentrations using a complete culture medium. The culture medium in the 96-well plate was discarded. In the drug treatment group, different concentrations of drug solutions were added, while the control group received complete culture medium. Both the drug treatment group and the control group were set up with 4 wells each, and 170 μl of drug solution or complete culture medium was added to each well. After 72 h, the drug solution and complete culture medium were discarded from the 96-well plate. 100 μl of diluted CCK8 solution [10% CCK8 (APE x BIO) + 90% RPMI-1640 (Gibco)] was added to each well, including the four blank wells without cells. After incubation at 37 ℃ for 3.5 h, the absorbance was measured at 450 nm using an enzyme-linked immunosorbent assay (BioTek, synergy H1). GraphPad Prism 8.0.2 software (GraphPad Inc., San Diego, CA, USA) was used to plot drug dose–response curves and calculate drug IC50. Experiments were repeated in triplicate.

#### In vivo tumorigenicity experiment

Cells in the logarithmic growth phase were trypsin digested, adjusted to a cell density of 1 × 10^7^/ml, and mixed evenly. Each NXG mouse was inoculated with 0.1 ml in the right shoulder. Tumor growth of nude mice was observed and recorded the next day. Four weeks later, the mice were euthanized and dissected to observe the growth of the transplanted tumor.

#### H&E and immunohistochemical staining

PDAC-X1 cells in the logarithmic growth phase were taken, enzymatically dissociated, and inoculated onto sterile glass slides. After 60 h, slides were washed with PBS, fixed with 4% paraformaldehyde (Servicebio) for 15 min, air-dried, and treated with 0.5% Triton X-100 (MCE) for 20 min. After paraffin embedding, the original tumor tissue and xenograft tumor were cut into 4 μm sections and dried at 60 ℃ for 5 h.

H&E staining: Prepared slices were stained with hematoxylin for 5 min, differentiated in hydrochloric acid and alcohol for 30 s, and then stained with eosin for 2 min. Finally, the slices were dehydrated and sealed with neutral resin.

Immunohistochemical staining: After dewaxing and rehydration, the slides were immersed in a sodium citrate solution (10 mmol/L, pH = 6.0), boiled for 90 s for antigen retrieval, and incubated in a 3% hydrogen peroxide solution at 37 ℃ for 15 min. 100 µl of normal goat serum dropwise was added to the slide, and sealed at 37 ℃ for 15 min. The slides were incubated with anti-CK7, anti-CK19, anti-E-cadherin, anti-Vimentin, anti-Ki-67, and anti-CA19-9 at 37 ℃ for 12 h, followed by the addition of secondary antibodies and incubation at room temperature for 50 min (Fuzhou Maixin ready-to-use antibodies). A DAB staining kit (Dako) was used for color development and rinsed with running water for 5 min. Slides were re-stained with hematoxylin, dehydrated with gradient ethanol, cleared with xylene, and sealed with neutral resin, before observation under an inverted microscope (Olympus, IX73 + DP74).

#### Statistical analysis

All statistical analyses were performed using the SPSS 26.0 software. The data were expressed as mean ± SD. Student’s t-tests and ANOVA were used for group comparisons. A *P*-value of < 0.05 was considered statistically significant.

## Results

### Establishment of PDAC-X1 cell line

Tumor cells isolated from the primary tumor were cultured for approximately six months to obtain a stable cell line, PDAC-X1. Under an optical microscope, PDAC-X1 displayed cell adherence and typical epithelial-like growth patterns with relatively uniform cell size. A small number of megakaryocytes and multinucleated cells were visible with large nuclei and distinct nucleolus were observed, consistent with the morphological characteristics of malignant tumors (Fig. 1C and D). Cellular morphology was stable and remained consistent despite repeated cycles of freezing and thawing.

### Analysis of DNA short tandem repeat sequences

**Fig. 2:**
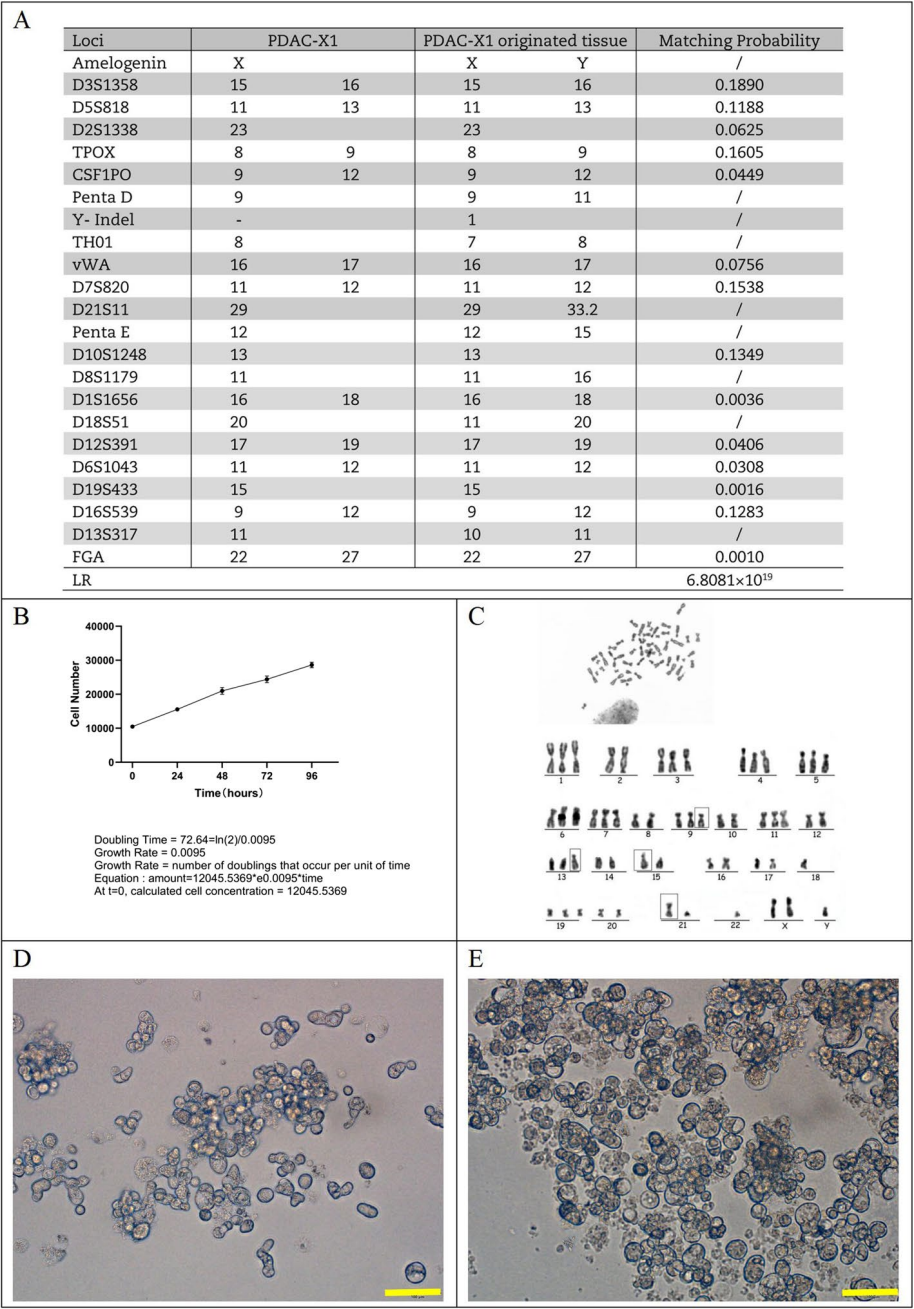
**A** STR results of PDAC-X1 and primary tumor; **B** Growth curve illustrating the doubling time of PDAC-X1 at approximately 73 h; **C** Karyotype analysis showed that PDAC-X1 was predominantly (90%) subtriploid, with representative karyotypes 55, XXY inv (9), 13p + , 15p + , rob (21,22); **D**-**E** PDAC-X1 organoid formation process. D: Organoid morphology three days post-inoculation; E: Organoid morphology 7 days post-inoculation. Scale bar = 100 μM

DNA typing analysis confirmed that the two submitted samples originated from the same individual with a likelihood ratio (LR) = 6.8081 × 10^19^ (Fig. 2A). This finding supports the conclusion that the PDAC-X1 cell line had the same genetic origin as the primary tumor tissue. Additionally, it did not match any existing profile in the ExPASy STR database, indicating PDAC-X1 as a novel human PDAC cell line.

### Cell growth curve

When cultured in RPMI-1640 medium supplemented with 10% fetal bovine serum, PDAC-X1 cells maintained a stable and rapid proliferation rate, with a population doubling time of 73 h (Fig. 2B).

### Chromosome analysis

Karyotype analysis showed that PDAC-X1 cells had complex karyotypes with significant variations in chromosome number and morphology. The majority of cells exhibited subtriploid karyotype (90%), whereas the remaining 10% of cells were characterized as subtetraploid. The representative karyotypes were 55, XXY inv (9), 13p + , 15p + , rob (21,22) (Fig. 2C).

### Tumor organoid culture

Inoculation of PDAC-X1 cells onto an ultra-low adsorption culture plate under complete medium conditions revealed that cells exhibited high levels of proliferation and formed organoids with small branching structures that increased in volume over time (Fig. 2D and E).

### Scanning electron microscopy and transmission electron microscopy

**Fig. 3:**
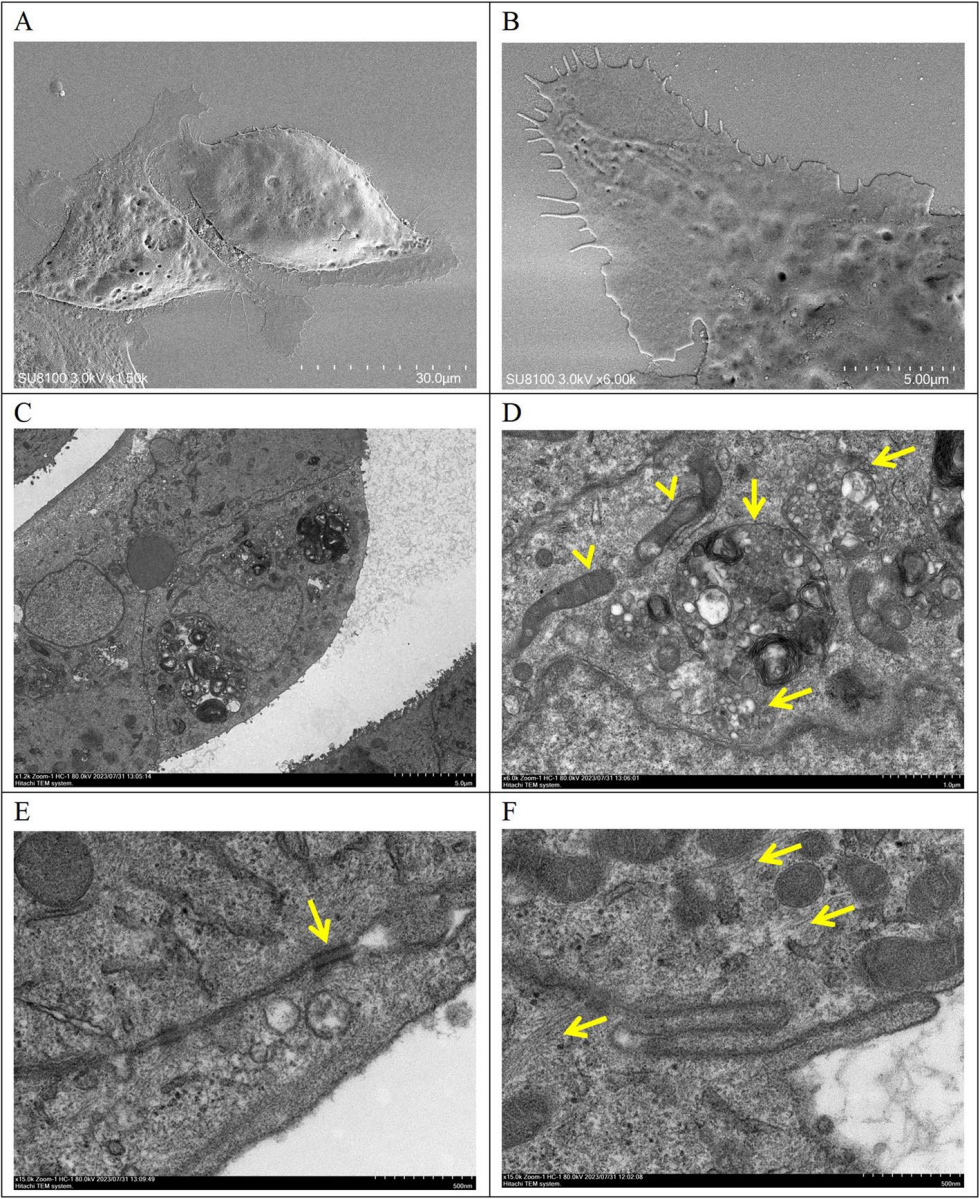
**A**-**B** PDAC-X1 cell morphology under scanning electron microscopy: **A** PDAC-X1 is mainly short shuttle and polygonal in shape; **B** A small number of microvilli and filamentous pseudopodia can be seen on the surface of PDAC-X1 cells; **C** Under transmission electron microscopy, the surface of PDAC-X1 cells has sparse microvilli, large nuclei, and wrinkled nuclear membranes; **D** PDAC-X1 has a relatively abundant number of organelles, mild swelling of mitochondria(yellow arrowhead), and numerous lysosomes visible(yellow arrow); **E** Visible desmosomes between PDAC-X1 cells(yellow arrow); **F** PDAC-X1 cells contain a significant amount of actin fibers(yellow arrow)

Scanning electron microscopy showed that PDAC-X1 cells had variable morphological features. Cells exhibited predominantly spindle-like or polygonal-like structures with varying sizes, limited cell surface microvilli, and visible filamentous pseudopodia (Fig. 3A and B). Transmission electron microscopy revealed that the cell surface consisted of sparse microvilli and enlarged nuclei with wrinkled nuclear membranes. Cells contained relatively abundant organelles, slightly swollen mitochondria, lysosomes, and desmosomes were visible between cells, as well as numerous actin fibers (Fig. 3C-F).

### Drug sensitivity experiment

**Fig. 4:**
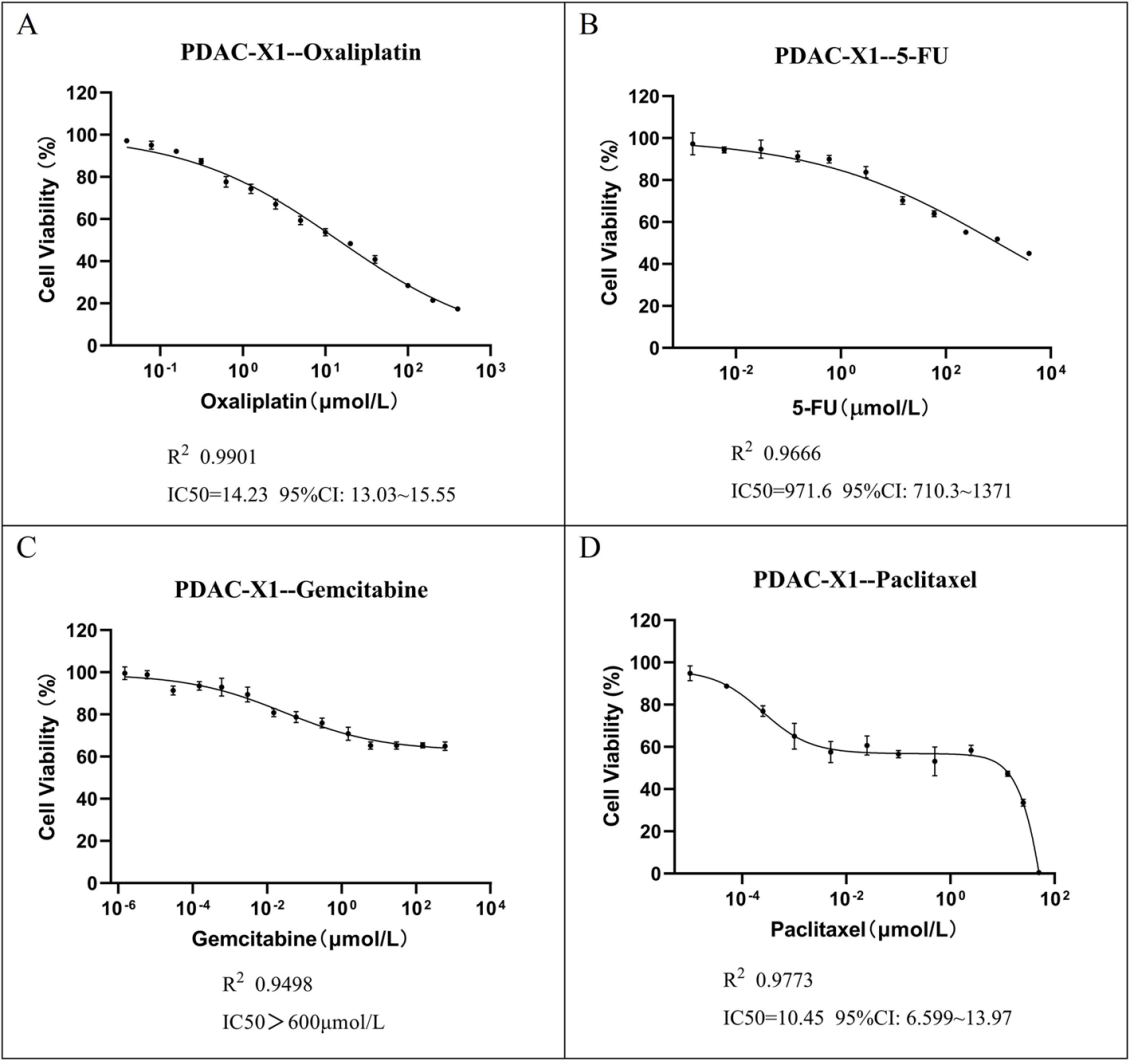
PDAC-X1 demonstrated resistance to **A** oxaliplatin, IC50 = 14.23µmol/L; **B** fluorouracil, IC50 = 971.6µmol/L; **C** gemcitabine, IC50 > 600 µmol/L; **D** paclitaxel, IC50 = 10.45µg/mL

PDAC-X1 exhibited resistance to multiple drugs, including oxaliplatin (IC50 = 14.23 µmol/L), fluorouracil (IC50 = 971.6 µmol/L), gemcitabine (IC50 > 600 µmol/L), and paclitaxel (IC50 = 10.45 µg/mL) (Fig. 4A-D). PDAC-X1 has been characterized as an intrinsic multidrug-resistant pancreatic cancer cell line.

### In vivo tumorigenicity experiment

**Fig. 5:**
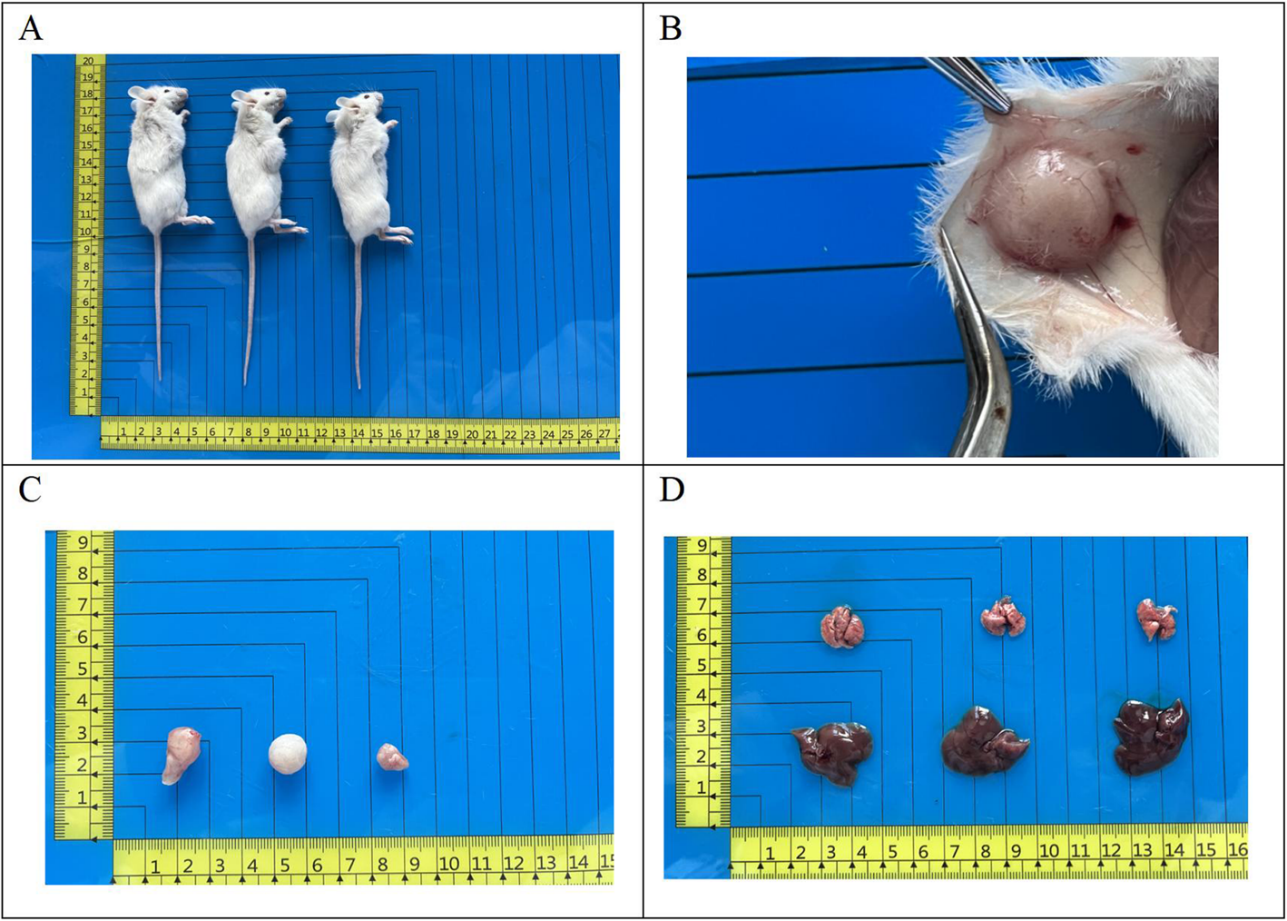
**A** After subcutaneous inoculation, PDAC-X1 rapidly formed transplanted tumors in NXG mice, with a tumor formation rate of 100%; **B** Gross view of transplanted tumors in vivo; **C** Gross view of transplanted tumors in vitro; **D** No metastatic transplanted tumors were observed in the lungs and liver of mice after one month of transplantation

Inoculation of 1 × 10^6^ PDAC-X1 cells subcutaneously into three NXG mice resulted in the formation of transplanted tumors with a tumor formation rate of 100% within 4 weeks. No metastatic lesions were found in the liver and lungs (Fig. 5A-D).

### H&E and immunohistochemical staining

**Fig. 6:**
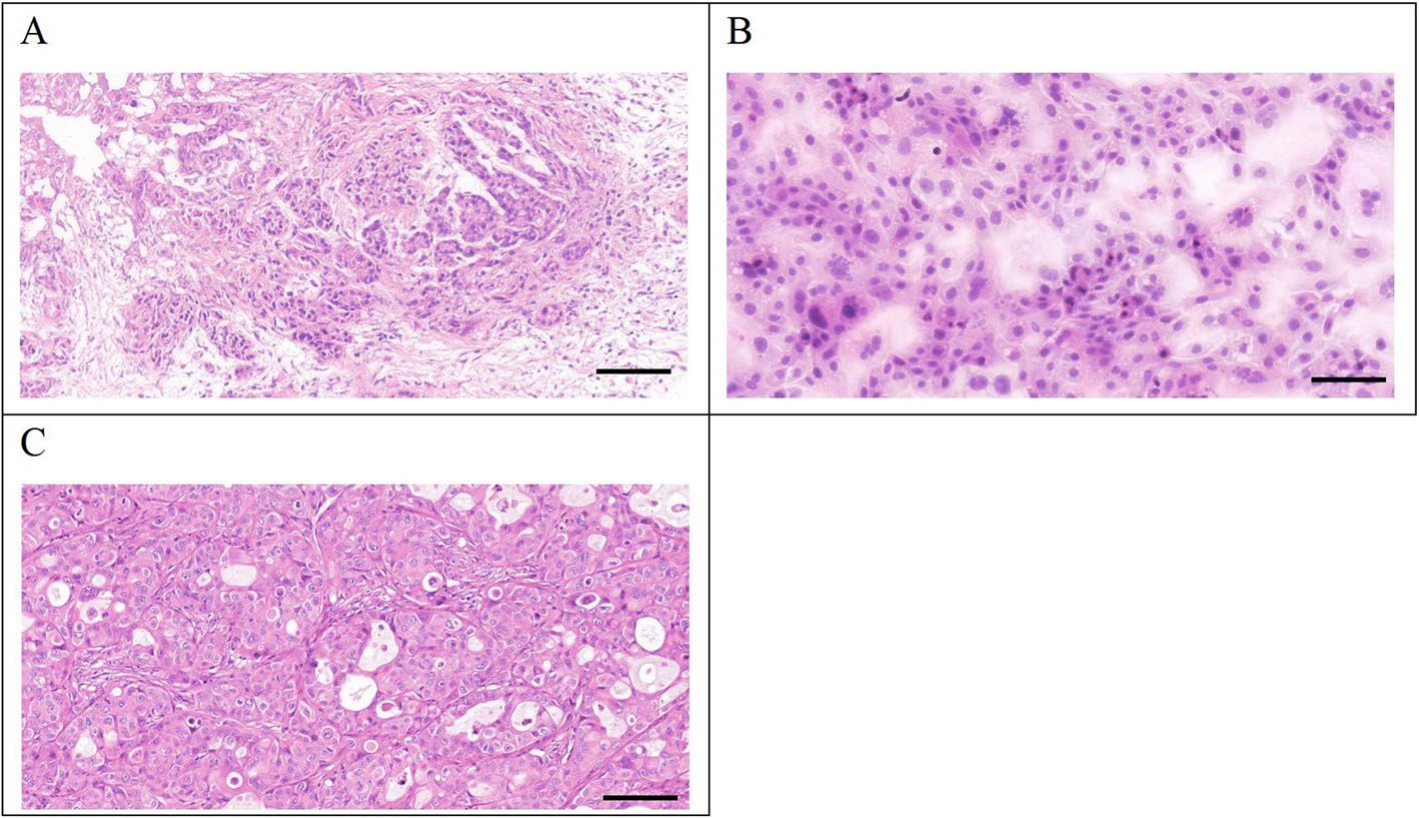
**A** Poorly differentiated primary tumor with tumor cells arranged in an irregular glandular tubular and nested-like, with abundant interstitial components surrounding the tumor. **B** PDAC-X1 cell size was relatively uniform, with enlarged nuclei, prominent nucleoli, and reduced cytoplasm. Polynuclear and megakaryocytes were visible, characteristic of malignant tumor cell manifestations. **C** Transplanted tumor cells exhibited irregular glandular-like structures, and the tissue structure resembled the primary tumor. Scale bar=50 μM

The post-operative pathological diagnosis of the patient's primary tumor showed poorly differentiated PDAC with irregular glandular tube-like and nest-like patterns and abundant stromal components surrounding the neoplastic cells (Fig. 6A).

H&E staining showed that PDAC-X1 cells were relatively uniform in size, with enlarged nuclei, prominent nucleoli, and reduced cytoplasm. Polynuclear and megakaryocytes were visible, characteristic of malignant tumor cell manifestations (Fig. 6B).

H&E staining of the transplanted tumor showed the formation of irregular glandular-like structures, resembling the histological morphology of the primary patient-derived tumor (Fig. 6C).

**Fig. 7:**
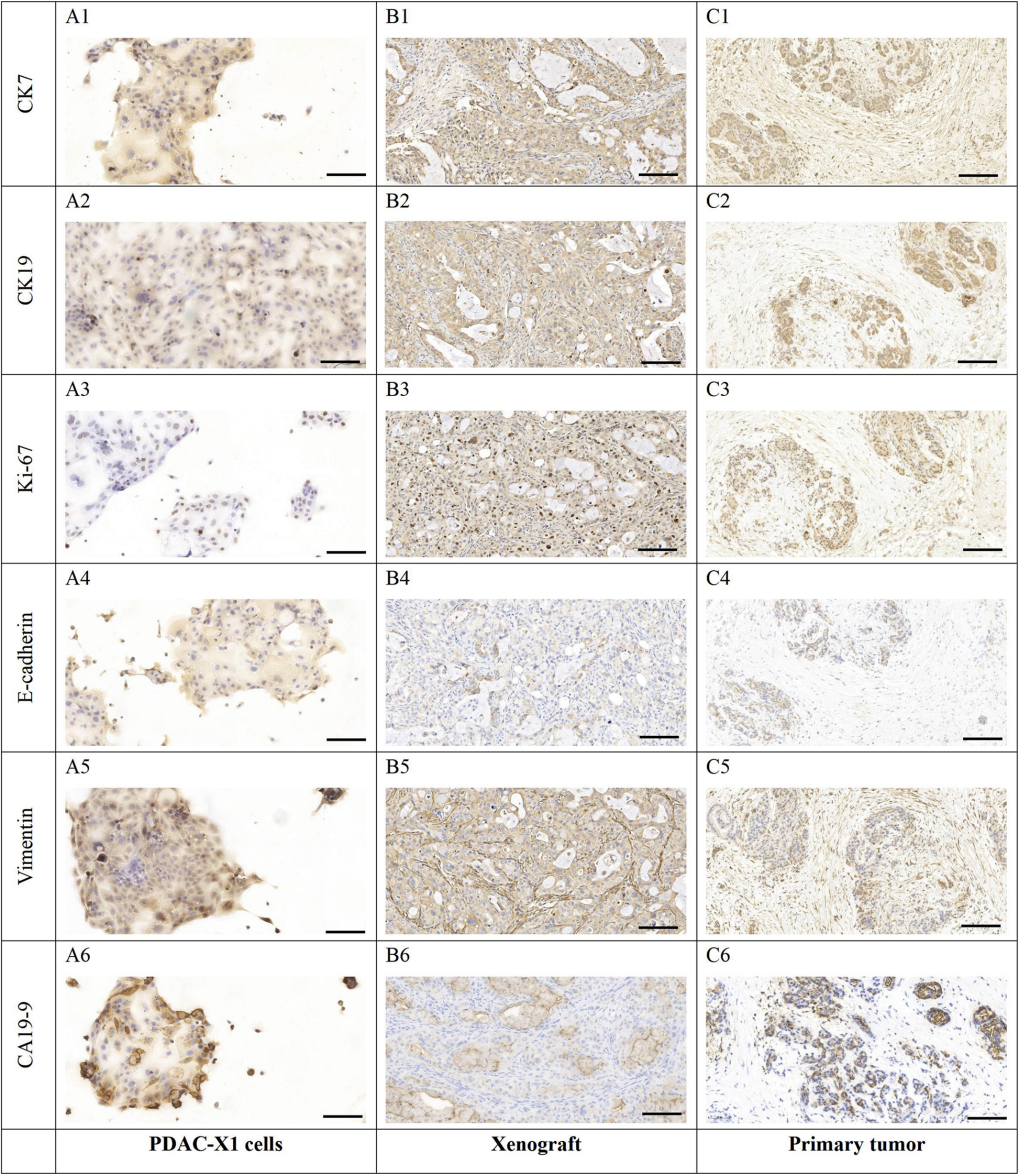
A1-A6 and C1-C6: In cell lines, transplanted tumors, and primary tumors, there was a positive expression of A1-C1: CK7; A2-C2: CK19; A3-C3: Ki-67, with an expression rate of 40%; A4-C4: E-cadherin; A5-C5: Vimentin; A6-C6: CA19-9. Scale bar = 50 μM

Immunohistochemistry showed positive expression of CK7 (Fig. 7A1-C1), CK19 (Fig. 7A2-C2), E-cadherin (Fig. A4-C4), Vimentin (Fig. 7A5-C5), and CA19-9 (Fig. 7A6-C6) in cell lines, transplanted tumors, and primary tumors. Ki-67 (Fig. 7A3-C3) ratio was 40%, consistent with rapid tumor proliferation.

## Discussion

Pancreatic cancer is predicted to become the second leading cause of cancer-related mortality by 2030. Tumor cell resistance to chemotherapy drugs, which can be innate or acquired, is a main factor contributing to ineffective cancer treatment [15]. Many PDAC patients initially respond to chemotherapy. However, cellular heterogeneity and the complex TME contribute to the rapid development of resistance, leading to treatment failure. A deeper understanding of the molecular mechanisms underlying PDAC progression and metastasis, as well as the interactions within TME, are crucial to elucidate the etiology and pathobiology of chemotherapy resistance and develop innovative combination therapies [16–18]. In this study, a novel cell line, PDAC-X1, was derived from the primary tumor tissue of Chinese PDAC patients and successfully established. Drug sensitivity tests confirmed that PDAC-X1 cells had intrinsic multidrug resistance that serves as an excellent model to investigate the underlying mechanisms of PDAC drug resistance.

Carbohydrate antigen (CA) 19–9 is considered the most specific and sensitive PDAC prognostic biomarker. Increased CA19-9 levels have been associated with tumor progression [19–21], and elevated preoperative levels have been linked to increased risk of recurrence and mortality in patients [22]. However, a subset of patients with a Lewis antigen-negative phenotype secrete little to no CA19-9, resulting in challenges to prognosis [23, 24]. In this study, the PDAC-X1 cell line and the corresponding transplanted tumors exhibited increased CA19-9, consistent with the elevated preoperative CA19-9 levels observed in the patient from whom the PDAC-X1 cells were derived. This finding suggests that PDAC-X1 recapitulates the CA19-9 environment of the original tissue, and therefore represents a valuable tool for the investigation of the mechanisms underlying CA19-9 and PDAC.

Chromosomal aneuploidy in tumor cells has been related to the increased incidence, progression, and poor prognosis of PDAC [25–27], as well as the development of drug resistance. Triploidy has been associated with endogenous drug resistance, whereas tumors exceeding tetraploidy have been associated with acquired drug resistance [28]. Karyotype analysis showed that subtriploid was the predominant karyotype of PDAC-X1, accounting for 90% of the cells, which may be one of the key factors contributing to the innate multidrug resistance exhibited in PDAC-X1.

Autophagy is a highly conserved, lysosome-dependent degradation mechanism that plays a protective role in enabling tumor cells to overcome adverse microenvironments and evade the damaging effects of radiation and chemotherapy. The process maintains intracellular homeostasis by isolating cytoplasm components and protein aggregates for degradation and recycling. In neoplastic tumors, this process sustains tumor cells and promotes growth. Autophagy satisfies high metabolic demands through macromolecular digestion and supports anti-apoptosis, dormancy, and multidrug resistance [29–32]. Transmission electron microscopy observations revealed increased lysosomes and proenzyme particles in PDAC-X1 cells, indicating active autophagy. Increased autophagic activity may be another key driver contributing to intrinsic multidrug resistance.

EMT has been associated with PDAC tumor invasiveness, metastasis, and multidrug resistance [33–35]. The downregulation or loss of E-cadherin and upregulation of vimentin are considered important markers of the EMT process [36–38]. The results showed that PDAC-X1 cells exhibited decreased E-cadherin coupled with increased vimentin, indicative of EMT phenotype. The EMT-like characteristic may be an additional factor contributing to the intrinsic multidrug resistance of PDAC-X1.

Mouse tumor models serve as powerful tools for studying tumor occurrence, progression, and evaluation of drug efficacy. However, some cell lines are incapable of forming transplanted tumors after inoculation into nude mice [39–41]. In the present paper, inoculation of PDAC-X1 cells into NXG mice led to the rapid formation of subcutaneous transplant tumors, with a short incubation period and a tumor formation rate of 100%. H&E examination showed PDAC-X1 transplanted tumor cell morphology closely resembled tissue of the primary tumor, indicating its usefulness as an in vivo experimental model.

Of course, this study only identified and analyzed the establishment and biological characteristics of a new pancreatic cancer cell line PDAC-X1, which may limit the universal application of this study in a wider population of PDAC patients. The subsequent establishment of more new pancreatic cancer cell lines and their sequencing analysis may deepen our understanding of the drug resistance mechanism of pancreatic cancer.

In conclusion, a novel intrinsic multidrug resistance human pancreatic cancer cell line, PDAC-X1, derived from a Chinese patient was established and characterized. PDAC-X1 represents a valuable experimental model to investigate the underlying mechanisms of drug resistance, as well as the development of novel therapeutic strategies for pancreatic cancer.

## Data Availability

The datasets used and/or analysed during the current study are available from the corresponding author upon reasonable request.

## Declarations

### Key resources

**Table:**

REAGENT or RESOURCE	SOURCE	IDENTIFIER
Software and algorithms		
GraphPad Prism 8.0.2	Grappa Inc	https://www.graphpad.com/
ImageJ1.52e	NIH	https://imagej.nih.gov/ij
“Cell calculator + + ” tool	V. Roth MD, Doubling Time Calculator (2006)	https://doubling-time.com/compute_more.php
